# Comparison of Prostate-Specific Promoters and the Use of PSP-Driven Virotherapy for Prostate Cancer

**DOI:** 10.1155/2013/624632

**Published:** 2013-01-31

**Authors:** Yi Lu, Yu Zhang, Guimin Chang, Jun Zhang

**Affiliations:** ^1^Department of Pathology and Laboratory Medicine, The University of Tennessee Health Science Center, Cancer Research Building, Room 218, 19 South Manassas Street, Memphis, TN 38163, USA; ^2^Department of Urology, University of Tennessee Health Science Center, Memphis, TN 38163, USA

## Abstract

Prostate cancer is the most frequently diagnosed cancer and the second leading cause of cancer deaths in men today. Although virus-based gene therapy is a promising strategy to combat advanced prostate cancer, its current effectiveness is limited partially due to inefficient cellular transduction *in vivo*. To overcome this obstacle, conditional oncolytic viruses (such as conditional replication adenovirus (CRAD)) are developed to specifically target prostate without (or with minimal) systemic toxicity due to viral self-replication. In this study, we have analyzed and compared three prostate-specific promoters (PSA, probasin, and MMTV LTR) for their specificity and activity both *in vitro* and *in vivo*. Both mice model with xenograft prostate tumor model and canine model were used. The best PSP was selected to construct a prostate-specific oncolytic adenovirus (CRAD) by controlling the adenoviral E1 region. The efficacy and specificity of CRAD on prostate cancer cells were examined in cell culture and animal models.

## 1. Introduction

Prostate cancer is the most frequently diagnosed malignancy and the second leading cause of cancer deaths in American men today with an estimated 241,740 new cases of prostate cancer and 28,170 deaths estimated in year 2012 [1]. Although virus-based gene therapy is a promising strategy to combat advanced prostate cancer, its current effectiveness is limited partially due to inefficient cellular transduction by therapeutic viral vectors *in vivo*. To overcome this obstacle, conditional oncolytic viruses (such as conditional replication adenovirus (CRAD)) are developed to specifically target prostate without (or with minimal) systemic toxicity due to viral “oncolytic” self-replication. 

The adenovirus early region 1 (E1) gene, which comprises E1a and E1b, encodes the viral early proteins that are necessary for adenoviral replication and the consequent oncolysis of permissive host cells. E1-deleted (including E1a-deleted) adenoviruses are replication defective and are commonly used as viral vectors to carry therapeutic genes for gene therapy. Replication-competent viruses, also known as oncolytic viruses, replicate within transduced cells and force these cells into a lytic cycle. Released virus is then able to infect neighboring cells until all susceptible cells are eliminated. Theoretically, a large tumor burden could be effectively eradicated using a small dose of an oncolytic virus. Therefore, strategies to use conditional oncolytic virus, or the so-called attenuated replication-competent viruses, to specifically target prostate tissue have been developed [2–4]. The idea behind this study is to place the Ad5 E1 region in *cis* complementation (i.e., use E1 as a transgene) back into an E1-deleted, replication-defective adenovirus under the control of a prostate-specific promoter (PSP). Thus, E1 protein expression will be confined strictly to the prostate tissues and render this a conditional oncolytic virus (CRAD) within the prostate. 

A number of PSPs have been defined over the years that include, but not limited to, promoters of prostate-specific antigen (PSA), probasin (PB), mouse mammary tumor virus (MMTV LTR), prostate-specific membrane antigen (PSMA), human glandular kallikrein 2 (hK2), and prostatic steroid-binding protein C3. Among them, we have extensively studied three of them: PSA, PB, and MMTV promoters. PSA is a protease that is, for all practical purposes, exclusively expressed in prostate [5]. PSA is overproduced by prostate cancer and elevated serum PSA level correlates with the volume of prostate cancer burden [5–7]. Circulating PSA-positive cells are also strongly associated with metastatic prostate cancer [8]. By using an expression vector, a 680 bp PSA promoter isolated from a prostate cancer patient demonstrated the prostate-specific expression of the reporter gene [9]. The PSA promoter has an androgen response element (ARE) and its activation requires both the androgen receptor (AR) and its ligand, androgen, to be present. PB, a rat prostate protein, is also expressed selectively in prostate [10]. The rat PB gene promoter (454 bp) contains ARE [11] and directs a hormonal and developmental regulated expression, of a heterologous gene, specifically to the prostate in transgenic mice [12]. Transgenic mice bearing PB promoter fused to simian virus 40 large tumor antigen gene all consistently develop prostate adenocarcinoma [13]. Similarly, the MMTV LTR has specificity for both breast and prostate tissues as determined by studies in both cultured cells and transgenic mice [14–16]. Retroviruses carrying MMTV LTR-antisense c-myc effectively inhibit the growth of prostate cancer DU145 cells *in vivo* [17]. MMTV LTR-TGF*β* transgenic mice have transgene expression and biological changes that are exclusive to the breast in female mice [18, 19] and prostate in male mice [20]. The 1.1 kb MMTV LTR appears to have higher prostate-specific expression of transgene than that of full-length MMTV LTR in transgenic mice [5]. 

In this study, a systemic comparison of three PSPs (a −650/+30 bp PSA, a −426/+28 bp PB, and a Cla I truncated 1.1 kb MMTV LTR) activity and specificity in the canine and mouse models followed by a PSP-driven E1-mediated oncolytic approach on prostate cancer has been conducted. We compared PSA, PB, and MMTV LTR for their promoter activity and tissue specificity side by side in canine and mouse models, as well as in both prostate and nonprostate cells *in vitro* and *in vivo*. The best PSP among the three, the PSA promoter, was selected to drive Ad5 E1 region to generate a prostate-specific CRAD, AdPSAE1. The efficacy and specificity of AdPSAE1 as a potential therapeutic vector for prostate cancer gene therapy were analyzed.

## 2. Materials and Methods

### 2.1. Construction of Adenoviruses Containing Prostate-Specific Promoter (PSP) Fused to lacZ

 PB/SV40t is an expression vector containing a 454 bp 5′ upstream region of PB gene [12]. To make AdPBlacZ, a 3.2 kb lacZ (*β*-galactosidase) gene containing a nuclear localization signal at the 5′ upstream was released from plasmid pPD1.27 (a derivative of pPD16.43 [21]) by Hind III and Dra I and ligated to PB/SV40t which had been cut by EcoR V, so that lacZ was placed downstream of the 456 bp PB promoter. The resultant plasmid was cut by Pst I and Apa I to release a PB-lacZ-polyA cassette. After polishing the ends, the cassette was ligated to an E1a-deleted adenoviral shuttle vector (Genetic Therapy Inc., Gaithersburg, MD, USA), whose endogenous RSV promoter had been removed, to generate the resultant recombinant adenoviral shuttle vector pPBlacZ. 

 A 680 bp 5′ upstream region (−650/+30) of PSA gene and a Cla I-truncated 1.1 kb MMTV LTR were used as PSA promoter and MMTV promoter. Adenoviral shuttle vectors pMMTVlacZ and pPSAlacZ were generated by replacing PB promoter in pPBlacZ with MMTV LTR and PSA promoter, respectively. Briefly, pPBlacZ was cut by Sal I and Xba I; the released lacZ shuttle vector backbone was ligated to a 1.1 kb MMTV promoter which was derived from pMAMneo (Stratagene, Lo Jolla, CA, USA) by cut with Cla I and Nhe I. The resultant recombinant adenoviral shuttle vector was pMMTVlacZ. A 650 bp PSA PCR product was formed by PCR using plasmid containing 5′ upstream sequence of PSA gene as template and two primers which were specific to the PSA 5′ upstream region and also introduced two restriction sites, Sal I and Xba I, one at the end of each primer, respectively. The 650 bp fragment of PSA promoter was purified and cut with Sal I and Xba 1, then ligated to pPBlacZ whose PB promoter was removed by Sal I and Xba I. The resultant recombinant shuttle vector was pPSAlacZ. All the structures of recombinant adenoviral shuttle vectors were confirmed by DNA sequencing. 

**Figure 1:**
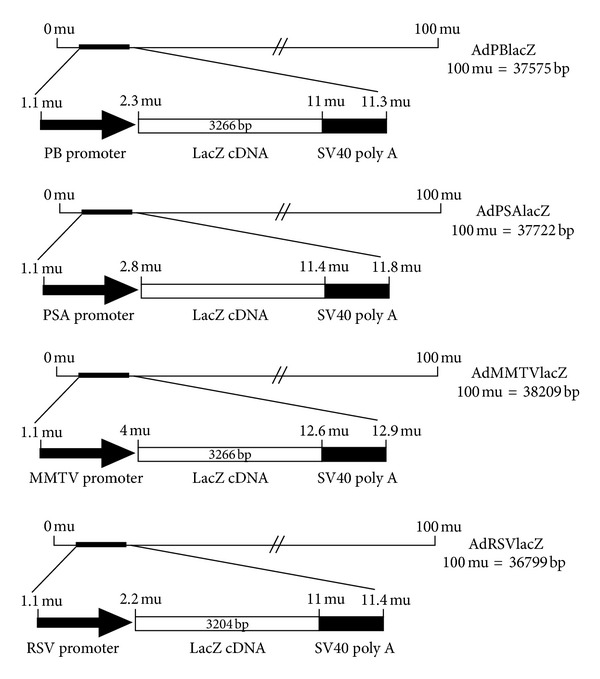
Schematic diagrams of various recombinant adenoviral vectors Ad-lacZ. The frame structure of adenoviral vectors expressing *β*-galactosidase (lacZ) under the control of prostate-specific promoters (AdPBlacZ, AdPSAlacZ, and AdMMTVlacZ) and constitutive promoter (AdRSVlacZ) is shown.

 Recombinant adenovirus AdPBlacZ, AdMMTVlacZ, and AdPSAlacZ were generated via *in vivo* recombination in 293 cells by the cotransfection of pJM17, an adenoviral genome plasmid, with the corresponding shuttle vectors pPBlacZ, pMMTVlacZ, and pPSAlacZ, respectively. The individual adenoviral plaques were screened by direct plaque-screening method as described previously [22]. By the similar way, AdRSVlacZ, in which lacZ gene was under the control of a constitutive Rous Sarcoma virus (RSV) promoter, was generated [23]. The schematic diagrams of these four adenoviral vectors were illustrated in Figure 1. Single viral clones were propagated in 293 cells and purified by BD Adeno-X Virus Purification Kits (BD Biosciences, Palo Alto, CA, USA). The viral titers were determined by plaque assays in 293 cells [24].

### 2.2. Delivery of Adenoviral Vectors to the Prostate of the Canine Model

Using a canine model, each of the adenoviral vectors of AdRSVlacZ, AdPBlacZ, AdPSAlacZ, and AdMMTVlacZ (4.8 × 10^9^ pfu each to one dog) was diluted in 1 mL of 0.9 M saline and delivered to one anesthetized dog (average age of one and half years old and average weight of 22 kg) by intraprostatic injection. Briefly, laparotomy was performed and 0.25 mL viral solution was directly injected into each quadrant of the prostate (4 × 0.25 mL = 1 mL total). At 72 hr, the dogs were sacrificed and various organs (prostate, liver, lung, spleen, bladder, brain, heart, kidney, external and internal iliac arteries, gonads, and vas deferens) were obtained at necropsy. The animal protocol was approved by the institutional IACUC. 

### 2.3. *β*-Galactosidase (*β*-Gal) Activity Assay

Prostate tissue was homogenized in 100 mL per 50 mm^3^ sample of 1x lysis buffer (*β*-Gal Assay Kit, Invitrogen, Carlsbad, CA, USA). After microcentrifugation at 14,000 rpm for 5 min at 4°C, the supernatant was collected. The protein concentration was determined by Coomassie Plus Protein Assay Reagent (Pierce, Rockford, IL, USA). The colorimetric *β*-galactosidase assay was performed by using *β*-gal Assay Kit (Invitrogen) according to the Manufacturer's protocol.

### 2.4. Polymerase Chain Reaction (PCR) for Detecting Viral Dissemination in Various Organs and Tissues

Genomic DNA was isolated using QIAamp Tissue Kit (Qiagen, Clarita, CA, USA) from organs and tissues according to the Manufacturer's protocol. The primers were specific for adenoviral type 5 genome and resulted in an 860 bp PCR amplification fragment within the homologous recombination region [25]. Primer 1 was 5′-TCGTTTCTCAGCAGCTGTTG-3′ and Primer 2 was 5′-CATCTGAACTCAAAGCGTGG-3′. PCR was performed in a 50 mL volume containing 250 ng DNA, 2 mM MgCl_2_, 50 mM KCl, 0.2 mM each of dNTPs, 20 mM Tris-HCl (pH 8.4), 2 mM each of the primers, and 2.5 units of Taq DNA polymerase (Gibco BRL). The reaction was carried out at 94°C for 5 min, then for 30 cycles at 94°C for 30 sec, 56°C for 30 sec, and 72°C for 1 min, followed by 72°C for 10 min.

### 2.5. Reverse-Transcriptase-Polymerase Chain Reaction (RT-PCR) for Detecting Transgene Expression

The total RNA was isolated using RNeasy Total RNA Kit (Qiagen) from organs and tissues according to the Manufacturer's protocol. After the treatment of total RNA with RNase-free DNase I (Gibco BRL), reverse-transcriptase reaction was carried out using Superscript II RT (Gibco BRL) according to the Manufacturer's protocol. An aliquot of the RT mixture was subsequently used for the PCR reaction. The primers were specific to *E. coli* lacZ gene and resulted in a 1036 bp PCR amplification fragment [26]. Primer 1 was 5′-GCCGACCGCACGCCGCATCCAGC-3′ and Primer 2 was 5′-CGCCGCGCCACTGGTGTGGGCC-3′. PCR was performed in 50 mL total volume containing 5 to 10 *μ*L above RT mixture, in a final concentration of 4 mM MgCl_2_, 50 mM KCl, 0.2 mM each of dNTPs, 20 mM Tris-HCl (pH 8.4), 2 mM each of the primers, and 2.5 units of Taq DNA polymerase (Gibco BRL). The reaction was carried out at 94°C for 4 min, then for 30 cycles at 94°C for 1 min, 60°C for 2 min, and 72°C for 2 min, followed by 72°C for 10 min. 

### 2.6. Southern Blot

The standard Southern blot transfer of PCR or RT-PCR cDNA products from agarose gel to Nylon membrane (Hybond-N^+^ Amersham Life Science, Buckinghamshire, UK) was performed as previously described [6]. The 860 bp and 1036 bp PCR products (see above) from positive control plasmids were purified and used as the probe for PCR and RT-PCR Southern, respectively. The probe was labeled by a-^32^P-dCTP using random primer method (Prime-It II Kit, Stratagene, La Jolla, CA, USA). The membrane was hybridized with the probe in Rapid-hyb buffer (Amersham Life Science) according to the Manufacturer's protocol. The membrane was exposed to a Kodak X-ray film between two intensifying screens at −80°C for autoradiography. 

### 2.7. Cell Culture and Medium 

Dulbecco's modified Eagle medium (D-MEM) was purchased from Gibco BRL (Gaithersburg, MD, USA). RPMI 1640 medium and McCoy's 5*α* medium were purchased from Cellgro (Herndon, VA, USA). Fetal bovine serum (FBS) was from Hyclone Laboratories (Logan, UT, USA). All cell lines were purchased from ATCC (Rockville, MD, USA) and were grown in D-MEM with 10% heat inactivated FBS. The human prostate cancer cell lines PPC-1 and LNCaP, both secret PSA (Dr. J. Norris of MUSC, personal communication), were grown in RPMI 1640 medium with 10% FBS. The human breast carcinoma MCF-7 cells and human bladder cancer RT4 cells were grown in McCoy's 5*α* medium with 10% FBS. Rat gliosarcoma 9L cells were grown in D-MEM medium with 10% FBS. All cells were grown in medium containing 100 units/mL penicillin, 100 ug/mL streptomycin at 37°C in a 5% CO_2_ atmosphere.

### 2.8. Adenoviral Transduction *In Vivo* on Xenograft Tumors

Xenograft tumors were established by injecting 5 × 10^6^ various cancer cells subcutaneously into the flank of each male Balb/c athymic nude mice (Harlan Sprague Dawley, Inc., Indianapolis, IN, USA). When tumors reached about 50 mm^3^ volume, 5 × 10^9^ pfu AdRSVlacZ and 1 × 10^9^ pfu or 1 × 10^10^ pfu prostate-specific Ad-lacZ were injected directly onto tumor site. The mice were euthanatized in 3 days and the tumors and other organs were harvested and sections were prepared. The animal protocol was approved by the institutional IACUC.

### 2.9. X-Gal Staining of Xenograft Tumors Transduced by lacZ Adenoviruses

Tumors samples were fixed in 4% paraformaldehyde for 30 min, then in 30% sucrose in PBS at 4°C until the samples sank to the bottom of the vial. The samples were then snap-frozen in liquid nitrogen in O.C.T. medium (Tissue-Tek/Sakura, Torrance, CA, USA) and processed to cryosections by a Cryostat. The cryosections were fixed in formalin for 30 sec then processed for X-gal staining as a measure of lacZ expression as described in [27]. For tumor whole-mount staining, tumors were fixed in 4% paraformaldehyde for 30 min, washed with PBS three times, and incubated overnight at 37°C in 1 mg/mL X-gal, 10 mM potassium ferricyanide, 10 mM potassium ferrocyanide, 0.01% sodium deoxycholate, 0.02% NP40, and 2 mM MgCl_2_ in PBS. The stained tumors were then post-fixed for 24 hr in 2.5% glutaraldehyde, 1% formaldehyde, and 0.1 M sodium deoxycholate (pH 7.0) in PBS. 

### 2.10. Analysis of Potential Oncolytic Effects of AdPSAE1 on Various Cell Lines

Cells (5 × 10^4^ per well) were plated in six-well plates; the next day the cells were either untreated or transduced with AdPSAlacZ or AdPSAE1 at the multiplicity of infection (moi) of 1. In brief, adenoviral infection of the cell lines was carried out by the addition of the viral solutions to cell monolayers and incubation at 37°C for 3 h with brief agitation every 15 min during the first 90 min incubation. After 3 h exposure to virus, the viral solution was deleted and a fresh medium was added to the cells. After 6 days of transduction, the medium was removed and the plates were washed twice with PBS. The wells were then completely covered with 2 mL of 1% crystal violet (Sigma, St. Louis, MO, USA) and the plate was allowed to sit 5 min with gentle rocking. After washing with water, the plate was allowed to dry at room temperature overnight before it was photographed.

### 2.11. *In Vitro* Growth Inhibition Assay by AdPSAE1

Cells (5 × 10^4^ per well) were plated in six-well plates; the next day the cells were divided into three groups: (a) control uninfected, (b) control virus AdPSAlacZ infected, and (c) AdPSAE1 infected. After viral infection.

### 2.12. *In Vivo* Tumor Growth Inhibition by AdPSAE1

PPC-1 cells (1 × 10^7^ cells in 0.2 mL of PBS) or RT4 cells (5.7  ×10^6^ cells in 0.2 mL of PBS) were injected subcutaneously into the flank of male Balb/c athymic nude mice (Harlan Sprague Dawley, Indianapolis, IN, USA). For each tumor cell model, three groups of mice were formed with 8 mice in each group. Group I was used as an untreated control. Group II and group III were for intratumoral viral injection of AdPSAE1 and control virus AdPSAlacZ, respectively. When tumors reached about 200 mm^3^ volume, a single dose of 5 × 10^6^ pfu AdPSAE1 or AdPSAlacZ was injected directly into each tumor mass. Tumor volume was measured every 3 days until the animals were sacrificed. All of the animals were sacrificed at day 35 after viral injection, when mice showed distress or had tumor burdens >15% of their total body weight.

## 3. Results

### 3.1. Study of PSP Activity and Specificity in Canine Model

To compare the specificity and activity of three different PSP (PSA, PB, and MMTV LTR promoters), we constructed replication-defective adenoviruses containing lacZ reporter gene under the control of PSA, PB, and MMTV promoters, respectively (Figure 1). In addition, we also constructed lacZ virus (AdRSVlacZ) under the control of the nonspecific, constitutively active RSV promoter for comparison. 

**Figure 2:**
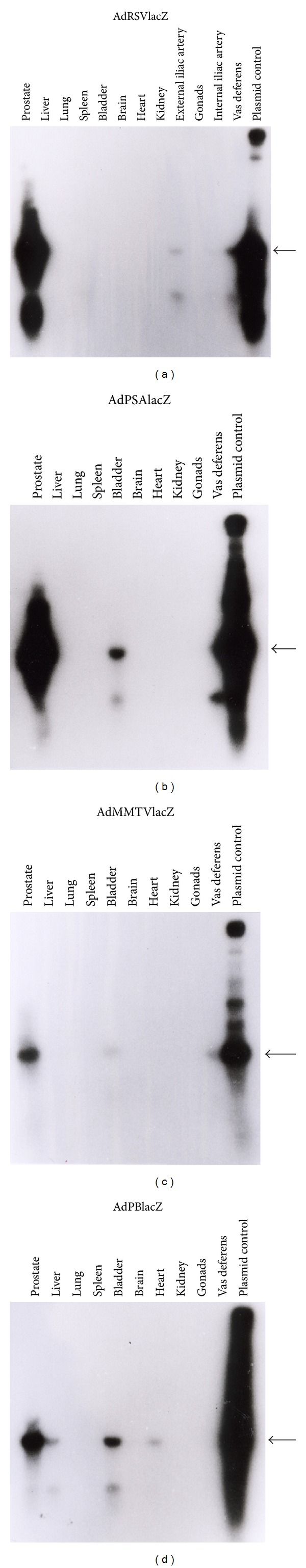
Adenoviral DNA sequence PCR Southern hybridization. DNA extracted from prostates and various organs at necropsy were subjected to PCR using primers specific to Ad5 genome. The expected PCR product of adenoviral sequences was a 860 bp band (shown by arrows). The PCR gel was transferred to a Nylon membrane by the Southern blot and the blot was hybridized with labeled probe which was the purified 860 bp PCR product from control adenoviral plasmid. Shown are the PCR Southern blots of dogs injected intraprostatically by AdRSVlacZ (a), AdPSAlacZ (b), AdMMTVlacZ (c), and AdPBlacZ (d).

 First, the activity and expression of reporter gene lacZ driven by these PSP were compared *in vivo* using a canine model by direct intraprostatic injection of these Ad-lacZ vectors. At 72-hour postinjection, various other organs including the prostate were harvested. To determine whether adenovirus dissemination occurred following intraprostatic injection, DNA was extracted from various canine tissues and was subjected to PCR Southern blot analysis for adenoviral sequences by using PCR primers flanked an 860 bp sequence of adenoviral genome [23]. We found that all adenoviral vector-injected prostates had an intense 860 bp signal band on agarose gel (not shown), confirming that the majority of adenoviral transduction occurred in the prostate. Interestingly, adenoviral sequences were found in tissues other than the prostate, suggesting that there was adenoviral dissemination following intraprostatic injection. For example, control viral vector AdRSVlacZ disseminated to the vas deferens (perhaps as a result of reflux from the ejaculatory ducts of the injected prostate) and external iliac artery. AdPSAlacZ and AdMMTVlacZ were also detected in vas deferens as well as in bladder tissues. Intraprostatic injection of AdPBlacZ resulted in adenoviral vector dissemination to the bladder, heart, and liver (Figure 2). Nevertheless, the majority of adenoviral vector DNA was found in the prostate following the intraprostatic viral injection. Adenovirus may disseminate to other organs and tissues, primarily bladder and vas deferens. The presence of adenoviral DNA in heart, blood vessels, and liver tissues may be explained by the fact that these organs overall receive a higher percentage of cardiac output (blood flow) than other organs. 

**Figure 3:**
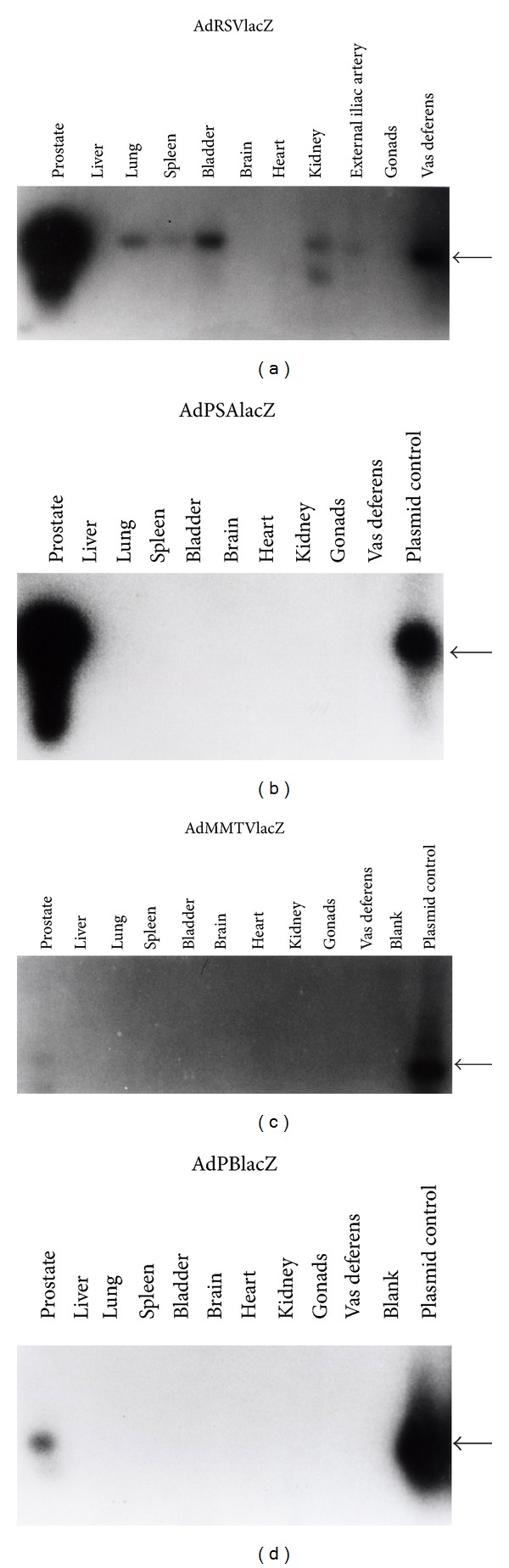
Southern of RT-PCR to determine lacZ mRNA expression. RNA extracted from prostates and various organs at necropsy were subjected to RT-PCR using primers specific to *E. coli* lacZ gene. The expected RT-PCR product was a 1036 bp band (shown by arrow). The RT-PCR gel was transferred to a Nylon membrane and the blot was hybridized with a labeled probe which was the purified 1036 bp PCR product from lacZ plasmid. Shown are the RT-PCR Southern blots of dogs injected intraprostatically by AdRSVlacZ (a), AdPSAlacZ (b), AdMMTVlacZ (c), and AdPBlacZ (d).

 Next, in order to determine the specificity of the PSP, we examined whether transgene lacZ was expressed in tissues containing adenoviral DNA. Total RNA was extracted from various canine tissues and subjected to RT-PCR analysis. The primers used for RT-PCR were specific for *E. coli* lacZ and flanked a 1036 bp internal sequence of the lacZ gene. Southern hybridization of RT-PCR gel (Figure 3) showed that a 1036 bp band was present in prostates following intraprostatic adenoviral injection. The control viral vector AdRSVlacZ had lacZ mRNA expression in all tissues where adenoviral vectors had disseminated (compare Figures 3(a) and 2(a)). This was expected as RSV has nonspecific promoter activity. In contrast, AdPSAlacZ and AdMMTVlacZ had no expression of lacZ in vas deferens and bladder (compare Figures 3(b), 2(b), 3(c), and 2(c)). Similarly, AdPBlacZ lacked detectable lacZ mRNA transcripts in bladder, heart, and liver (compare Figures 3(d) and 2(d)). Taken together, the results of Figures 2 and 3 confirmed that although prostate-specific adenoviral vectors do disseminate following intraprostatic injection, the adenoviral lacZ transgene was expressed only in the prostate. Interestingly, some organs such as liver, lung, spleen, bladder, and kidney, which had no detectable adenoviral DNA sequences by PCR Southern, had lacZ mRNA expression by RT-PCR Southern hybridization, implying that RT-PCR of lacZ transgene may be more sensitive than PCR of adenoviral sequences (compare Figures 3(a) and 2(a)). The integrity and equality of the RNA samples from various canine tissues was confirmed by RT-PCR of the housekeeping gene *β*-actin from the same cDNA pools used for RT-PCR of lacZ (not shown). 

### 3.2. Study of PSP Activity in Canine Prostates and Xenograft Tumors in Mice

**Figure 4:**
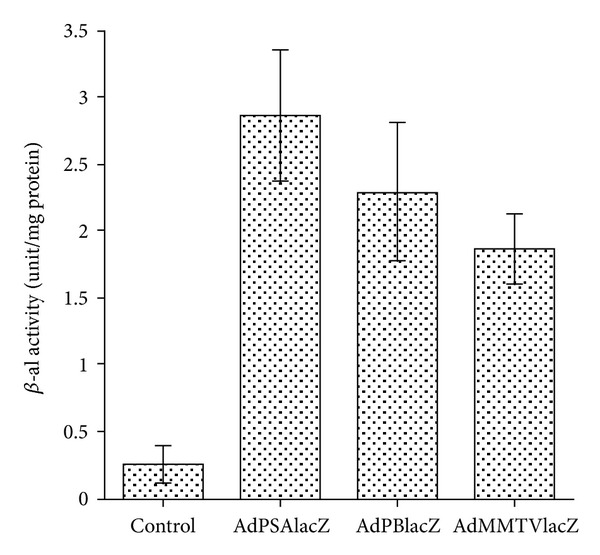
*β*-Galactosidase activity of canine prostate. Cell extracts from control and adenoviral intraprostatic injected canine prostates were isolated and *β*-galactosidase activity was assayed.

**Figure 5:**
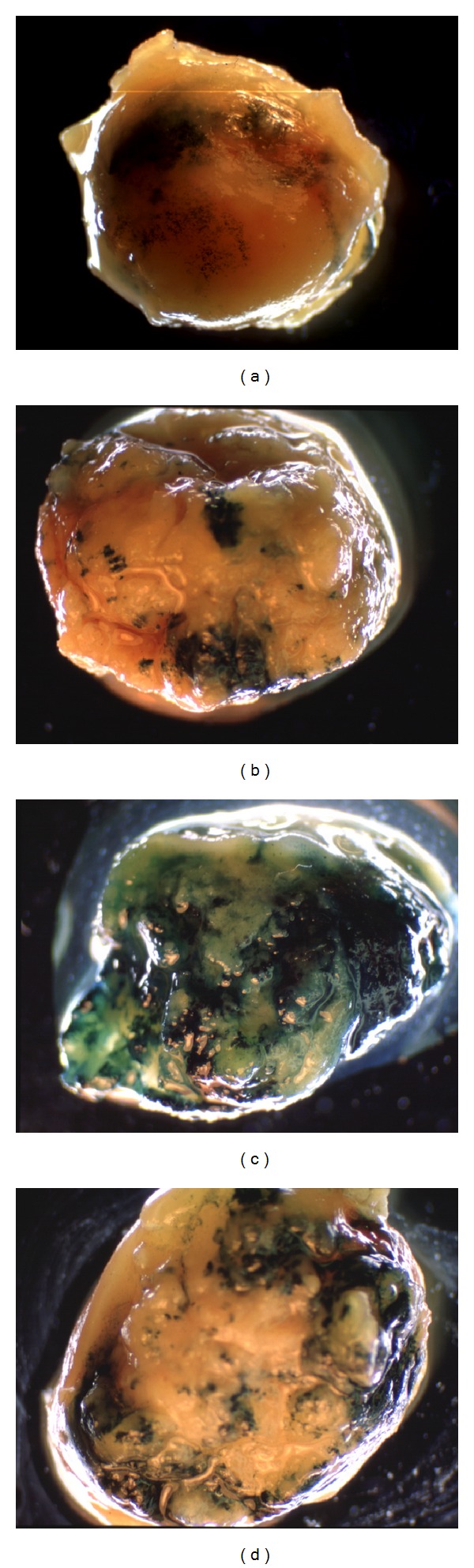
Comparison of *in vivo* activity of prostate-specific and RSV promoters. PPC-1 xenograft tumors were established and transduced by Ad-lacZ as described in Section 2. The tumors were harvested in 72 hr and processed to whole-mount tumor X-gal staining. Shown is X-gal staining of tumors transduced by 1 × 10^10^ pfu AdPBlacZ (a), 1 × 10^10^ pfu AdPSAlacZ (b), 1 × 10^9^ pfu AdRSVlacZ (c), and 1 × 10^10^ pfu AdRSVlacZ (d). Untreated control PPC-1 tumor did not show blue cells after X-gal staining (not shown).

The activity of these three PSPs was compared in the canine prostates after corresponding Ad-lacZ intraprostatic injection. PSA and PB promoters showed better PSP activity than MMTV LTR *in vivo*: protein extracts isolated from canine prostates were used for the colorimetric *β*-galactosidase assay to measure the lacZ enzymatic activity. Among the three PSP-driven Ad-lacZ, AdPSAlacZ had the highest activity (2.86 *β*-gal units/per mg protein), compared to AdPBlacZ (2.29 *β*-gal units/per mg protein) and AdMMTVlacZ (1.86 *β*-gal units/per mg protein) (Figure 4). However, the activity of the PSP is significantly lower than that of the constitutively active RSV promoter. As demonstrated by the whole-mount X-gal staining of the xenograft prostate tumors that grew in mice and were intratumoral injected with various Ad-lacZ viruses (Figure 5), the percentage of blue cells in tumors transduced by 1 × 10^10^ pfu AdPSAlacZ (Figure 5(a)) or 1 × 10^10^ pfu AdPBlacZ (Figure 5(b)) was much less than tumors transduced 1 × 10^10^ pfu AdRSVlacZ (Figure 5(c)). As a matter of fact, the level of PSP promoter activity was similar to that seen following injection by 1 × 10^9^ pfu AdRSVlacZ (Figure 5(d), in comparison with Figures 5(a) and 5(b)). This indicates that PSP had one log less promoter activity *in vivo* compared to that of constitutively active promoter such as RSV. 

### 3.3. PSA-Driven E1 Expression Effectively and Specifically Inhibits Prostate Cancer Cell Growth *In Vitro*

**Figure 6:**
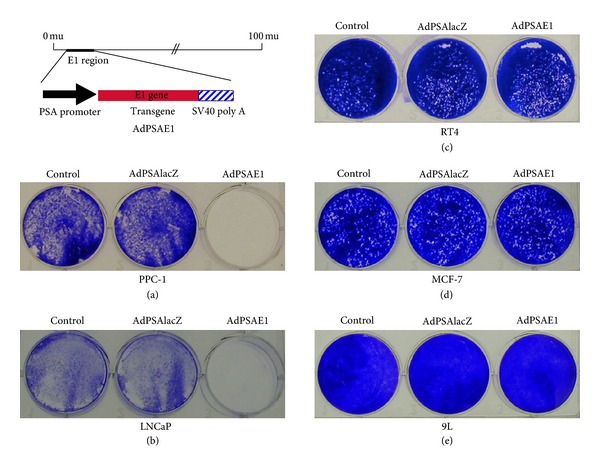
Conditional oncolytic effects of AdPSAE1 in prostate cancer cells. The design of a prostate-specific conditional replication-competent adenovirus AdPSAE1. The native Ad5 early region 1 (E1) gene that is required for adenoviral replication is replaced by an expression cassette that contains an Ad5 E1 gene under the control of an 860 bp PSA promoter. Human prostate cancer cell lines PPC-1 (a) and LNCaP (b), human bladder cancer cell line RT4 (c), human breast cancer cell line MCF-7 (d), and human glioma cell line 9L (e) were transduced with AdPSAE1 or AdPSAlacZ at moi of 1. Attached viable cells were stained with crystal violet 6 days after viral infection and were compared to the untreated controls.

As the 620 bp PSA promoter demonstrates the best promoter activity (and equal or better specificity) among the three PSP we analyzed (Figures 3 and 4), we have generated a prostate-specific, conditional oncolytic adenovirus, AdPSAE1 (Figure 6), by replacing the lacZ transgene of AdPSAlacZ (Figure 1); the resultant AdPSAE1 is exactly the same genome structure as AdPSAE1 except that the E1 gene has replaced lacZ gene, so that the PSA-E1 expression cassette replaces the native wild-type E1 region in Ad5 genome (Figure 6). As E1 is required and sufficient for adenovirus self-replication and consequent oncolysis in the host cells, accordingly, this strategy allows the expression of E1 protein under the control of a prostate specific promoter (PSA, as previously demonstrated by AdPSAlacZ in regulating lacZ expression shown above), enabling the adenovirus to replicate and enter the oncolytic cycle only in prostate cells. The antiprostate tumor efficacy and specificity of AdPSAE1 were examined *in vitro* and *in vivo* in both prostate and nonprostate cancer models.

The potential oncolytic cell-killing effects of AdPSAE1 were analyzed in various cancer cells. The human prostate cancer lines PPC-1 and LNCaP and nonprostate cancer cell lines RT4 (human bladder cancer), MCF-7 (human breast cancer), and 9L (human glioma) were infected with AdPSAE1 or control virus AdPSAlacZ at moi of 1. Viable cells were stained with crystal violet 6 days after infection and were compared to untreated control cells (Figure 6). As dead cells typically detach, crystal violet stains only those viable cells that remain attached to the culture dish. As shown in Figures 6(a) and 6(b), AdPSAE1 (right well) almost completely wiped out all PPC-1 and LNCaP cells, whereas AdPSAlacZ (middle well) had no cell-killing effects as compared to the untreated control (left well), respectively. On the other hand, AdPSAE1 had no cell-killing effects on RT4 (Figure 6(c)), MCF-7 (Figure 6(d)), and 9L (Figure 6(e)) cells. These results clearly demonstrate that AdPSAE1 selectively replicates (thus goes through the oncolytic cycle and kills the host cells) in cancer cells derived from the prostate (PPC-1 and LNCaP), but not in nonprostate cancer cells (RT4, MCF-7, and 9L). 

**Figure 7:**
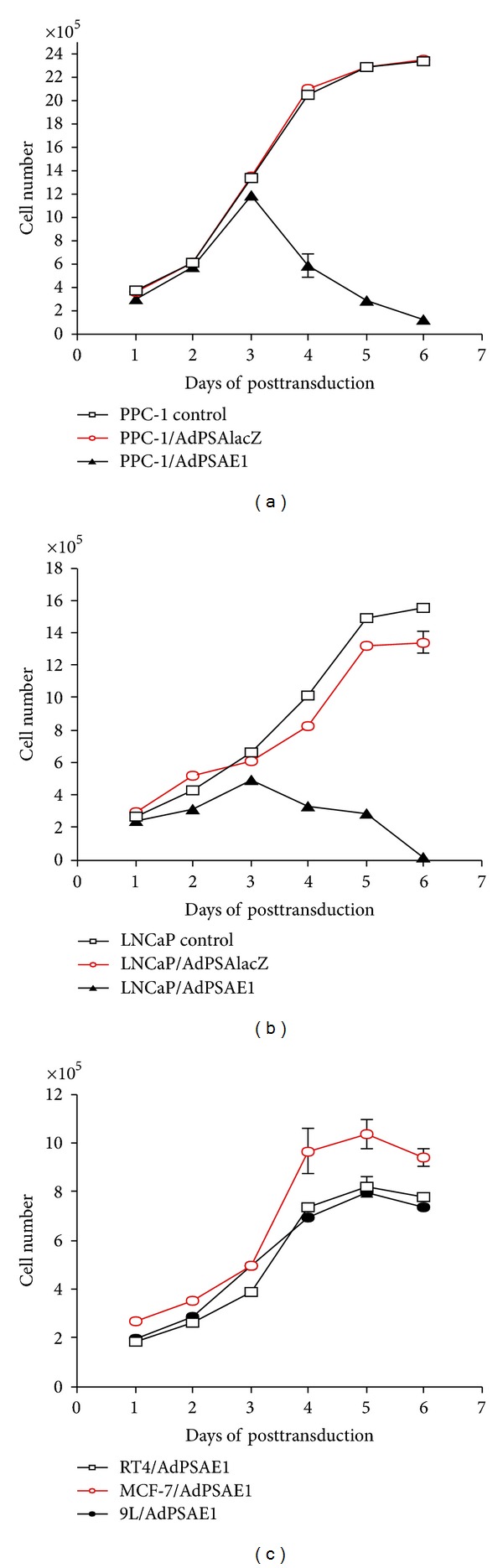
Time course of the growth inhibition effects of AdPSAE1 on prostate cancer cells. Prostate cancer cells PPC-1 (a) and LNCaP (b) and nonprostate cancer cells (RT4, MCF-7, and 9L) (c) were transduced with AdPSAE1 at moi of 1. Cell numbers were determined daily from day 1 to 6 after viral transduction. Untreated and AdPSAlacZ transduced cells were used as controls. The data represent the results from two independent experiments, each performed in duplicate. Some error bars are too small to show.

To analyze the time course of the growth inhibition effects of AdPSAE1 on prostate cancer cells, PPC-1 and LNCaP cells were either untreated or transduced with AdPSAE1 or control virus AdPSAlacZ at moi of 1 *in vitro*, and the cell numbers were monitored. As shown in Figure 7, significant growth inhibition was observed starting at day 4 after AdPSAE1 infection, with complete growth inhibition at day 6 for both prostate cancer cell lines PPC-1 and LNCaP. AdPSAlacZ transduction did not cause significant growth inhibition in either of these cell lines (Figures 7(a) and 7(b)). On day 6 after *in vitro* viral transduction at moi of 1, AdPSAE1 transduction significantly reduced the numbers of PPC-1 and LNCaP cells to 81.6% and 96.9% of untreated control values, whereas the control virus AdPSAlacZ transduction resulted in minor and insignificant growth inhibition (Figures 7(a) and 7(b)). In contrast, AdPSAE1 had no significant cell-killing or growth inhibition effects towards the nonprostate cancer cells RT4, MCF-7, and 9L when compared to the untreated control and control virus AdPSAlacZ transduced groups (Figure 7(c)). These results suggest that, *in vitro*, AdPSAE1 effectively leads to prostate-specific oncolytic killing.

To ensure that selective viral replication accounted for the cell killing in AdPSAE1 transduced cells, RT-PCR was performed using primers specific to Ad5 E1a gene and followed by Southern blot hybridization [23] to examine the E1a mRNA expression in AdPSAE1-transduced cells. We found that only LNCaP and PPC-1 cells had positive E1a RT-PCR product whereas RT4, MCF-7, and 9L cells did not (not shown), indicating that E1a was selectively expressed in prostate cancer cells. We also performed RCA (replication-complement adenovirus) assay by sequential infection of target cells (prostate and nonprostate cells) with AdPSAE1 and consequently collected the supernatant of target cells to infect 293 cells. We only found plaques in 293 cells infected by supernatant from PPC-1 and LNCaP cells that had been initially infected by AdPSAE1, not by supernatant from nonprostate cancer cells infected by AdPSAE1 (not shown). These results indicate that only AdPSAE1-transduced prostate cancer cells generate progeny viruses. 

### 3.4. Specific Expression of Transgene Driven by the PSA Promoter in the Xenograft Prostate Tumors in Animal Model

**Figure 8:**
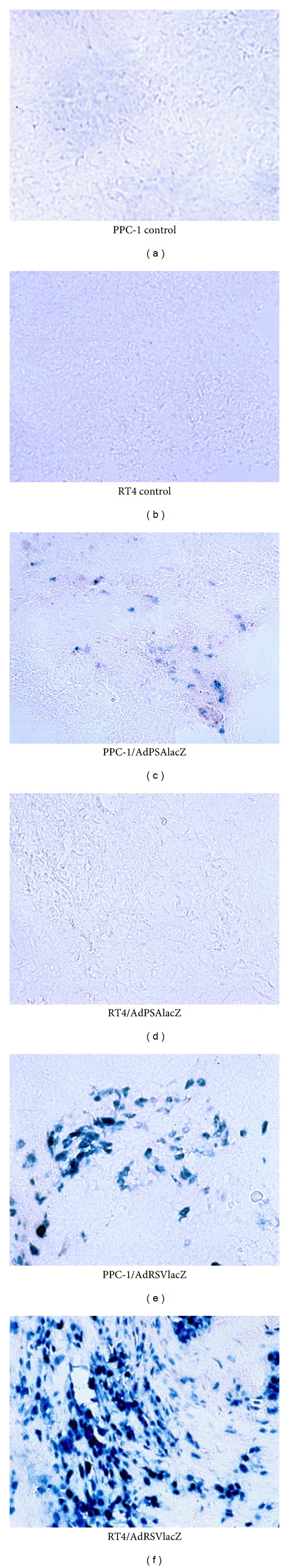
Specific transgene expression driven by a PSA promoter in prostate cancer cells. Xenograft tumors were established by subcutaneous injection of cancer cells into the flank of nude mice. When tumors reached about 50 mm^3^, each of the adenoviral constructs was injected directly into the tumor. The tumors were harvested 72 hr later and processed to cryosections. Shown is X-gal staining of tumor sections derived from prostate cancer PPC-1 cells ((a), (c), and (e)) and bladder cancer RT4 cells ((b), (d), and (f)). (a) and (b) are untreated control tumors to serve as negative controls. (c) and (d) are tumors transduced by AdPSAlacZ (1 × 10^10^ pfu). (e) and (f) are tumors transduced by AdRSVlacZ (5 × 10^9^ pfu) to serve as positive controls.

To demonstrate PSA-driven expression specifically in the prostate in the mouse model, we examine the specificity of prostate-specific promoters xenograft tumors from both prostate origin and nonprostate cancer cells were established in nude mice. A dose of 1 × 10^10^ pfu AdPSAlacZ was injected into subcutaneous xenograft tumors derived from human prostate cancer PPC-1 cells or human bladder cancer RT4 cells. As a positive control, AdRSVlacZ [23], an adenovirus containing the lacZ gene under the control of a constitutively active RSV promoter, was injected into xenograft tumors at a dose of 5 × 10^9^ pfu. LacZ expression was determined through X-gal staining of the cryosections of the tumors 72 h following viral injection. Untransduced control PPC-1 (Figure 8(a)) and RT4 (Figure 8(b)) tumors did not express detectable endogenous lacZ. AdPSAlacZ transduced PPC-1 tumors contained X-gal positive (blue stained) cells (Figure 8(c)), whereas AdPSAlacZ transduced RT4 tumors did not (Figure 8(d)). In contrast, both PPC-1 (Figure 8(e)) and RT4 (Figure 8(f)) tumors transduced by AdRSVlacZ showed X-gal positive cells. These results demonstrate that the expression of the lacZ transgene driven by this 680 bp PSA promoter occurred only in xenograft prostate tumors, but not in xenograft bladder tumors. However, the activity of the PSA promoter is much lower than that of the constitutively active RSV promoter (Compare Figures 8(c) and 8(e) with the blue stained cells and the viral dose injected, resp.). 

### 3.5. AdPSAE1 Specifically Inhibits Prostate Tumor Growth *In Vivo*

**Figure 9:**
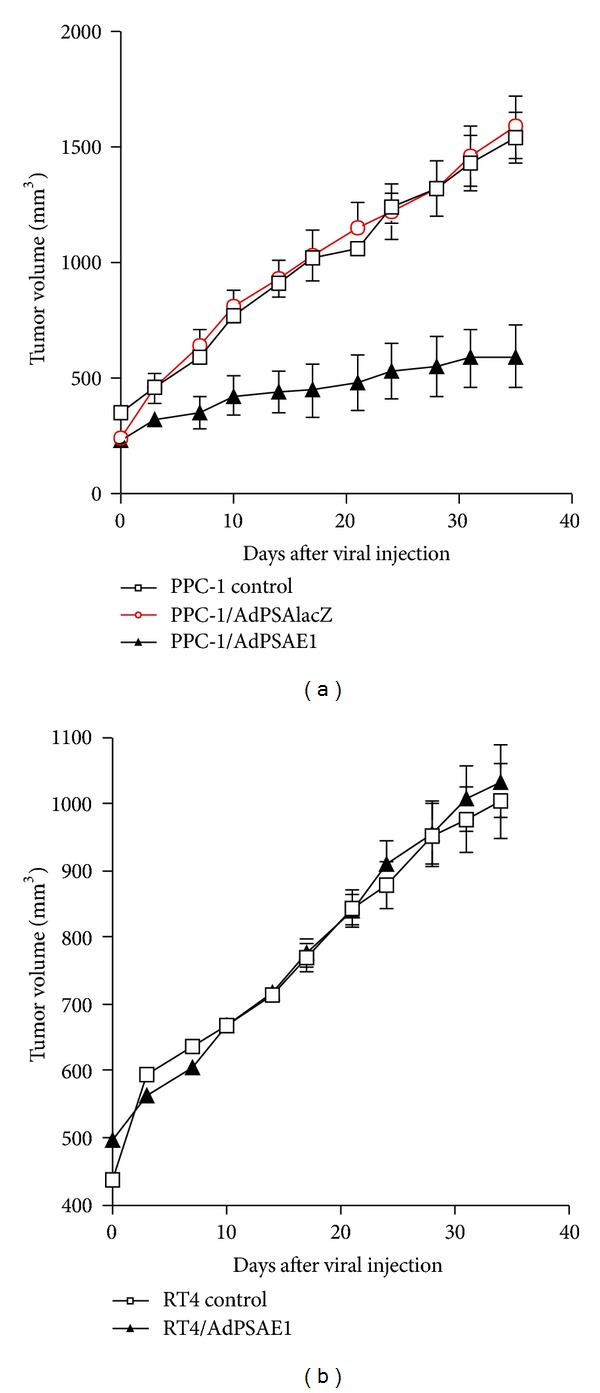
AdPSAE1 specifically inhibits prostate tumor growth *in vivo*. (a) The human prostate cancer line PPC-1 and (b) human bladder cancer line RT4 were injected subcutaneously into the flank of nude mice. When tumors reached an average volume of 200 mm^3^, tumors were either untreated (control) or treated with intratumoral injection (day 0) with 5 × 10^6^ pfu of AdPSAlacZ (control virus) or 5 × 10^6^ pfu AdPSAE1. The tumor sizes were periodically measured after viral injection. Each point represents the average tumor volume from 8 mice. Some error bars are too small to show.

To determine whether AdPSAE1 causes similar tumor growth inhibition *in vivo* as was shown *in vitro* (Figures 6 and 7), human prostate cancer PPC-1 cells and human bladder cancer RT4 cells were injected subcutaneously into the flank of nude mice to establish the xenograft tumors. When tumors developed to about 200 mm^3^, a single dose of AdPSAE1 was injected directly into the tumor in both cancer cell models. As shown in Figure 9(a) for the PPC-1 tumor model, both untreated tumors and tumors treated with control virus AdPSAlacZ grew rapidly and at a similar rate. In contrast, the AdPSAE1-treated group showed an effective suppression of this rapid growth. By day 35 after viral injection, the group treated with AdPSAE1 had a remarkable 61.8% reduction of tumor size as compared to the untreated group (Figure 9(a)). On the other hand, the same single dose of AdPSAE1 injected into the RT4 xenograft tumors failed to result in significant growth inhibition, as compared to the untreated RT4 tumor group (Figure 9(b)). The Western of tumor extracts confirmed that only PPC-1 tumors treated with AdPSAE1 showed detectable E1 protein expression (not shown). These results suggest that AdPSAE1 is able to specifically inhibit prostate tumor growth* in vivo*.

## 4. Discussion

To prevent unintended cytotoxic gene expression in nontargeted tissues during gene therapy (particularly when a suicide gene or oncolytic gene such as E1 is the expressed gene), tissue and/or cell-type specific promoters are required in order to tightly control the transgene expression. While it is ideal to have a cancer cell specific promoter to target diseased cells and spare the normal cells in the same organs, in the case when such a promoter is not available and the organ is not vital (as in the case of prostate cancer therapy), an organ/tissue-specific promoter would work as well. In this study, three PSPs were compared and the best one (PSA promoter) was used to control E1-mediated oncolytic therapy for prostate cancer. 

Most current gene therapy clinical trials are using viral vectors that are self-replication defective. These replication deficient viruses were designed for the safety reason in order to prevent viral oncolysis and replication in the host. These safe gene-transfer vehicles represent the early-stage gene therapy viral vectors which deliver therapeutic transgenes without exposing host cells to the danger of viral lytic cycle. However, the low transduction rate of viral vectors *in vivo* confines therapeutic transgene expression to only those cells along the injected needle track due to the viral inability to pass the transgene to neighboring cells. Consequently, the effectiveness of a therapeutic viral vector is directly correlated to its transduction efficiency. Although the bystander effect of certain therapeutic transgenes in the suicide gene therapy strategy for cancer helps to increase some therapeutic index, its effect is limited. Tumor cells cannot be 100% transduced with a single treatment. Untransduced tumor cells survive, divide, and eventually offset the therapeutic effects posed by the initial viral transduction. Therefore, repeated viral injections aimed at infecting those tumor cells not infected in the first round of viral transduction and those newly divided tumor daughter cells are required to achieve a successful gene therapy. However, due to the strong host immunogenic responses caused by adenoviral vectors, the second and subsequent rounds of adenoviral administration possess significantly reduced therapeutic effects *in vivo* [28, 29].

To overcome this obstacle, approaches that employ conditional oncolytic viruses, also called attenuated replication-competent viruses (such as CRAD), have been proposed for cancer gene therapy. The principle of conditional oncolytic viruses is that those viruses are engineered such that they can specifically target a desired cell type, or they are several orders of magnitude more susceptible to cause oncolytic cell lysis in the desired target cells than in the nontargeted cells. Our study showed that a 680 bp (−650/+30) PSA promoter is sufficient enough to drive a prostate-specific transgene expression in both canine prostates and xenograft tumors in mice. This PSA promoter-driven E1 expression led to a complete killing on prostate cancer cells *in vitro* and a significant inhibition of prostate tumor growth in mice with no toxic effects on nonprostate cells or tumors. 

In the study, we observed that the activity of PSA promoter, while maintaining its faithful tissue-specific expression, is noticeably weaker (by at least 10-fold) compared to the constitutive active RSV promoter (compare Figure 5(a) with 5(c) and Figure 8(c) with 8(e), resp.). This implies that, as a tradeoff for the tissue specificity, the expression of a therapeutic transgene driven by the PSA promoter will be much lower than that of a constitutively active promoter. Still, this may not present a major issue in our case because we are using an oncolytic strategy in which the therapeutic transgene, itself, is the Ad5 E1 gene. The relatively low degree of PSP activity may suffice, bring E1 expression to a level that causes oncolysis. Theoretically, only low levels of E1 expression are required to initiate and maintain the continuing viral oncolytic cycle to eradicate all prostate cells inside a tumor. In this study, we have demonstrated that at moi of 1, AdPSAE1 was able to completely eradicate all cancerous prostate cells *in vitro* (Figures 6 and 7). Similarly, in our *in vivo* study, at viral doses (i.e., intratumoral injection of 5 × 10^6^ pfu AdPSAE1 per tumor of 200 mm^3^ size, Figure 9) much lower than that of the typical E1-deleted adenoviral vectors we have routinely used (i.e., intratumoral injection of 5 × 10^9^ pfu E1-deleted adenovirus containing a therapeutic gene per tumor of 100 mm^3^ size, [30, 31]), AdPSAE1 exhibited an equivalent inhibition ability for xenograft prostate tumor growth as those by E1-deleted adenovirus at a much higher dose. However, we were still unable to completely eradicate tumors using AdPSAE1 treatment *in vivo* (Figure 9(a)). This may be partially due to the insufficient production of the E1 protein *in vivo* by the relatively weak prostate-specific promoter. 

The idea of using conditional oncolytic viruses for cancer therapy (virotherapy) is a very promising strategy that may make a large advance toward the optimal goal-complete eradication of primary tumor cells and the targeting of tumor metastases. While there are still a number of hurdles to overcome, one apparent emphasis for this oncolytic approach includes tissue/cell-type specific targeting in order to ensure safety; the other is targeting metastatic prostate cancer cells by systemic administration. A study to evaluate the biodistribution and toxicity of a replication-competent adenovirus following intraprostatic injection demonstrated that although the virus persisted in the urogenital tract and liver, most of the toxicity was minimal and self-limiting. Most importantly, there was no germ-line transmission of viral genes [32]. One way to control viral spread is to incorporate a prodrug enzyme gene in the CRAD, so the prodrug can be used as desired to suppress viral replication effectively. An example of that approach is the designing of a replication-competent, E1b-attenuated adenovirus containing CD/HSV-TK fusion gene; not only the suicide gene system allows for the utilization of double-suicide gene therapy, but also it provides a means to eliminate the virus and controls viral spread whenever needed [33]. Moreover, to target metastatic and circulating prostate cancer cells by systemic administration, tissue-specific promoters (such as PSP) can be combined to modified viral vectors with tropism to cancer cells to have a dual regulation control at the tissue-specific level (transcriptional targeting) and cell type-specific level (transductional targeting) [34, 35]. 

In addition to tissue-specific-promoter- (such as PSPs) driven oncolytic approach, tumor-specific promoters including survivin, carcinoembryonic antigen (CEA), and telomerase have also been used in regulated E1A expression in oncolytic adenoviral approach. Survivin overexpresses in several human tumors including gliomas [36] and prostate cancer [37]; thus surviving promoter is a good candidate for tumor-specific regulated E1a expression in gliomas [38, 39] and both PSA-producing and -nonproducing prostate cancers. Likewise, a CEA promoter-driven E1a replication-competent adenovirus, OV798, preferentially replicates in and kills CEA-producing colorectal cancer cells, but its replication is attenuated by 1000-fold in the CEA-negative cell lines [40]. The telomerase (hTR and hTERT) promoters are active in most cancer cells but not in normal cells, thus making the telomerase promoter-controlled E1a replication-competent adenovirus an attractive approach for tumor-specific oncolytic targeting [41]. Studies using telomerase-specific oncolytic viruses demonstrated their effective anti-tumor abilities both *in vitro* and *in vivo* [42–45]. In addition, the promoter of the CREBBP/EP300 inhibitory protein 1 (CRI1), a gene specifically expressed in malignant pleural mesothelioma, was used to drive E1a-mediated virotherapy that specifically kills malignant mesothelioma cells but not normal cells [46]. Other tumor-specific promoters used to drive E1-mediated virotherapy against various cancers include mucin-1 promoter [47], osteocalcin promoter [48], AFP promoter [49], midkine promoter [50], and COX-2 promoter [51, 52]. 

The ongoing efforts for a better understanding of the oncolytic cycle of viral biology and a continued development of cancer-specific conditional replication-competent viruses have brought hope closer to generating the ideal oncolytic virus for prostate cancer gene therapy. With significant improvement in viral transductional targeting and transcriptional regulation, we expect that oncolytic virus-based gene therapy will become a potential and effective means for prostate cancer treatment in the near future.
